# Monitoring of Psychotropic Medications in Children and Adolescents With Eating Disorder in a Community Setting

**DOI:** 10.1192/bjo.2026.11680

**Published:** 2026-06-30

**Authors:** Poornima Khadanga, Alla Pavlovic

**Affiliations:** 1 South London and Maudsley Trust, London, United Kingdom; 2 Northamptonshire Healthcare Foundation Trust, Northampton, United Kingdom

## Abstract

**Aims:**

Psychotropic medications are commonly used as an adjuvant to treat children having an eating disorder. Though there is little evidence on the rationale of use of psychotropic medications in eating disorder, they have often been used to address the associated psychiatriccomorbidities. While eating disorder owing to its complexities requires regular physical monitoring, introducing psychotropic medication makes it even more important for carrying out a regular monitoring. Hence the audit was designed as clinic practice mandatesits needs.

**Methods:**

Data collected from patient records in two different timelines- Dec 2022-Jan 2023 and Dec 2023-Jan 2024. Trust and NICE guidelines on monitoring antipsychotic medications and NICE guidelines for antidepressant monitoring were taken as standard guidelines.

**Results:**

31 patients were started on psychotropic medications in these time periods with most prescribed drug being Fluoxetine, followed by olanzapine and sertraline.It was seen that the compliance for physical health parameters (Blood Pressure, Heart Rate, BMI etc) was almost 100% except for waist circumference which was not being done on a regular basis. With the bloods, there was a good compliance on LFT, U&Es, FBC, Blood glucose levels but lipid profile, creatinine phosphokinase was not being routinely done for the young people

**Conclusion:**

The MEED monitoring is normally used in the eating disorder clinics which does not always coincide with the monitoring for psychotropics, for example - timing and/or type of investigations, therefore there is sometimes a gap in monitoring psychotropics. Waist circumference could be a difficult parameter to monitor because young people deal with body image concerns, hence this could be tricky to monitor. Based on the findings, we have designed a monitoring tool which the physical health team has been using from now. We are hoping to see better results when reaudit is done.

